# Cost-Effectiveness of Targeted Prophylaxis among Allogenic Stem Cell Transplant Recipients

**DOI:** 10.3390/ph16030466

**Published:** 2023-03-21

**Authors:** Nour Shbaklo, Costanza Vicentini, Alessandro Busca, Luisa Giaccone, Chiara Dellacasa, Irene Dogliotti, Tommaso Lupia, Carla M. Zotti, Silvia Corcione, Francesco Giuseppe De Rosa

**Affiliations:** 1 Department of Medical Sciences, Infectious Diseases, University of Turin, 10124 Turin, Italy; 2 Department of Public Health and Paediatrics, University of Turin, 10124 Turin, Italy; 3 Stem Cell Transplant Center, AOU Citta’ Della Salute E Della Scienza, 10126 Turin, Italy; 4 Unit of Infectious Diseases, Cardinal Massaia, 14100 Asti, Italy; 5 School of Medicine, Tufts University, Boston, MA 02111, USA

**Keywords:** antibiotic prophylaxis, bloodstream infections, onco-hematologic transplant, multi-drug resistant infections, cost-effectiveness analysis

## Abstract

Bloodstream infections (BSI) are life-threatening complications for onco-hematologic patients. Fluoroquinolones prophylaxis (FQP) was recommended for patients with neutropenia. Later, it was correlated with increased resistance rates among this population and its role became debated. While the role of FQ prophylaxis is still being studied, its cost-effectiveness is also unknown. The objective of this study was to evaluate the costs and effects associated with two alternative strategies (FQP vs. no prophylaxis) for patients with hematological malignancies undergoing allogenic stem cell transplant (HSCT). A decision-tree model was built integrating retrospectively collected data from a single transplant center, part of a tertiary teaching hospital in Northern Italy. Probabilities, costs and effects were considered in the assessment of the two alternative strategies. Probabilities of colonization, BSIs, extended-spectrum beta lactamase (ESBL) and *Klebsiella pneumoniae* carbapenemase (KPC) BSIs and mortality associated with infection, as well as median duration of length of stay (LOS) were calculated based on data collected between 2013 and 2021. The center applied the strategy of FQP between 2013 and 2016, and of no prophylaxis between 2016 and 2021. Data on 326 patients were collected during the considered time period. Overall, the rates of colonization, BSI, KPC/ESBL BSI, and mortality were 6.8% (95% confidence interval (CI) 2.7–13.5), 42% (9.9–81.4) and 20.72 (16.67–25.26), respectively. A mean bed-day cost of 132€ was estimated. Considering no prophylaxis vs. prophylaxis, the difference in costs ranged between additional 33.61 and 80.59€ per patient, whereas the difference in effects ranged between 0.11 and 0.03 life-years (LYs) lost (around 40 and 11 days). Given the small differences in terms of costs and effects between the two strategies, no prophylaxis seems an appropriate choice. Furthermore, this analysis did not consider the broader effect on hospital ecology of multiple doses of FQP, which could provide further support for the strategy of no prophylaxis. Our results suggest that the necessity for FQP in onco-hematologic setting should be determined based on local antibiotic resistance patterns.

## 1. Introduction

Bacterial bloodstream infection (BSI) is a common complication of neutropenia after chemotherapy in hematologic malignancies, causing high morbidity and mortality [1,2,3]. Immunosuppression due to underlying disease, chemotherapy, invasive procedures, antibiotic therapy and hospitalization increases the risk of infections [4,5]. Bacterial BSIs account for around 20 to 30% of febrile neutropenia cases in patients with hematologic malignancies [6,7]. This trend is also similar in several European and American reports [8,9,10].

In 2007, fluoroquinolones (FQs) prophylaxis was recommended by the European Conference on Infections in Leukemia (ECIL) for patients with neutropenia for more than 7 days [11,12]. Later, concerns for the rise of fluoroquinolone resistance and the lack of its effect on mortality in trials have challenged this practice [13]. In fact, association was reported between FQs use and emergence of extended spectrum beta-lactamase (ESBL) producing *Enterobacteriaceae* [14,15,16]. In a recent meta-analysis, fluoroquinolone prophylaxis had reduced mortality in neutropenic patients [17], whereas two large trials failed to demonstrate a similar benefit [18,19]. As such, variations exist between guidelines, with some supporting antibiotic prophylaxis [20] and others recommending against it [21]. Resistance selection is the most considered adverse event associated with prophylaxis. This phenomenon directed many international guidelines to recommend the monitoring of vancomycin-resistant *Enterococci*, methicillin-resistant *Staphylococcus aureus*, FQ-resistant Gram-negatives, and *Clostridioides difficile* infections, in order to decide prophylactic strategies upon local epidemiology. Besides selection of resistant strains, FQ might cause other adverse events, such as gastrointestinal, musculoskeletal, and neurologic symptoms, also as a result of interactions with other cancer drugs [11,17].

The recent re-evaluation of the topic by ECIL states possible benefits for FQ prophylaxis on BSI, though not on overall mortality. In addition, it is recommended that toxicity and changes in local epidemiology should be considered when applying FQ prophylaxis. Attention should now be drawn to reduce unnecessary antibiotic use in an attempt to preserve the activity of agents.

While effectiveness of FQ prophylaxis in oncology patients is recognized, its cost-effectiveness is still unknown. The objective of this study was to evaluate costs and effects associated with two alternative strategies (FQP vs. no prophylaxis) for patients with hematological malignancies receiving allogeneic hematopoietic stem cell transplantation (HSCT).

## 2. Materials and Methods

### 2.1. Study Design and Definitions

A decision tree model was used to compare the cost-effectiveness of fluoroquinolone prophylaxis (FQP) to no prophylaxis in preventing colonization, blood-stream infections (BSIs) and mortality [22]. The input parameters integrated data collected retrospectively from a single transplant center at a 1200-bed university hospital, Molinette, located in the City of Health and Science with primary and secondary referrals in Northern Italy. The center applied the strategy of FQP (intravenous levofloxacin 500 mg) between 2013 and 2016, and no prophylaxis between 2016 and 2021. All patients undergoing transplantation at the center during the considered time frame were included in the analysis. Patient and microbiological data were collected retrospectively. MDR rectal screening was performed weekly and blood cultures were performed when clinically suspected or in presence of symptoms.

Probabilities, costs (in terms of costs associated with length of stay (LOS), drug costs) and effects (in terms of life-years gained, LYs) were considered in the assessment of the two alternative strategies. Probabilities of colonization (rectal swab positive for ESBL or KPC), BSIs, one-year mortality as well as median duration of LOS were calculated based on data collected between 2013 and 2021. BSIs were classified as antimicrobial resistant (AMR) BSI or non-AMR BSI. AMR BSI was defined as bacteremia with ESBL or KPC. Patients did not receive anti-infective therapy when colonized without fever.

Patients received either FQP or no prophylaxis and transitioned along the arms of the decision tree according to the probability of three outcomes: colonization, infection, mortality. Every step in the sequence of events leading to an outcome was associated with a cost and terminal nodes were associated with an effect value in terms of LYs gained, resulting in a total cost and effectiveness value per strategy based on the likelihood of the sequence of events.

### 2.2. Microbiology Detection Methods

Identification of bacterial isolates and antimicrobial susceptibility testing (AST) were performed on the Microscan Walkaway 96 plus system (Beckman Coulter, Brea, CA, USA) according to the EUCAST breakpoints. ESBL phenotypes were detected according to the latest version of the “EUCAST guidelines for detection of resistance mechanisms and specific resistances of clinical and/or epidemiological importance”. It was detected by broth microdilution tests integrated into the Microscan Walkaway panels. ESBL production test was positive if a ≥8-fold reduction was observed in the MIC of cefotaxime, ceftazidime or cefepime in combination with clavulanic acid (fixed concentration 4 mg/L) in comparison with the MIC of cephalosporins alone.

Carbapenemase production was detected by the phenotypic modified Hodge test and defined or confirmed by the combination disk test (MAST, UK; Rosco, Denmark) until 2018; later immunochromatographic lateral flow tests (ICT) were used for rapid detection and typing of carbapenemases. Species identification was performed by MALDI–TOF MS.

### 2.3. Costs and Utilities

Unit costs were valued in euros from the hospital’s perspective. The total cost per strategy included the sum of the drug cost and hospital stay. BSIs costs were assumed identical for each branch (FQP vs. no prophylaxis), and were therefore excluded from the analysis. The hospital stay costs were calculated by multiplying the average cost of a day of hospital stay by the average number of days of hospitalization for each outcome. The average cost of a day of hospitalization was calculated on the basis of data from the onco-hematology ward.

Based on estimates from the literature [23], a 20.8% reduction on Italian life expectancy was applied to estimate the life expectancy for patients receiving allogeneic blood or marrow transplantation [24]. A 3% discounting rate was applied using the following discounting formula: Y_n_ = X^n^/(1 + r)^n^. Considering the median age of admitted patients, the number of LYs gained if patients survived was estimated to be 8.42 LYs, applying 3% discounting.

### 2.4. Statistical Analysis

**Figure 1:**
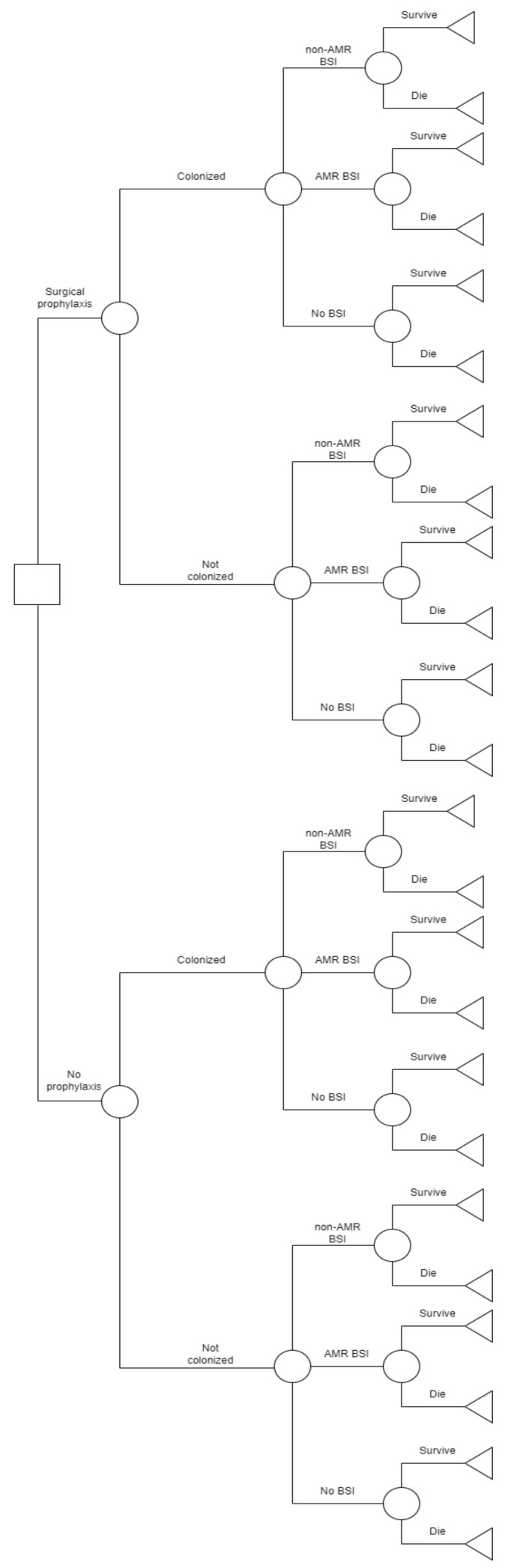
Decision tree model for FQP vs. no prophylaxis. AMR: antimicrobial resistance; BSI: bloodstream infections.

Chi-square tests were run to determine significant differences in colonization, BSI and mortality between the two strategies. A cost-effectiveness analysis was conducted, evaluating the outcomes as cost per LY gained. One-way sensitivity analysis was conducted to assess the impact of the uncertainty of the following input parameters: probability of colonization, BSI, AMR-BSI as well as mortality rate, over the range of the respective 95% confidence intervals (CI). Threshold values, beyond which a strategy would become cost-effective, were investigated. Decision tree models (Figure 1) were created using SilverDecisions 1.2.1 [25].

## 3. Results

Data on 326 patients was collected during the considered time period. No significant difference was outlined in colonization, BSI, AMR BSI in both strategies in univariate analysis. However, there was a significant difference in the 1-year mortality during the two periods; 30.5% in FQP period and 0% in the no FQP period (*p* < 0.001). The cost of FQP was 0.57 euros per dose (500 mg). Overall, the rates of colonization, BSI, KPC/ESBL BSI and mortality were 6.8% (IC 95% 2.7–13.5), 42% (9.9–81.4) and 20.72 (16.67–25.26), respectively. Input parameters are detailed in Table 1. The median LOS was 32 (IQR 29–37.75), 35 (30–44) and 35.5 days (32–43) for patients with no infection, BSI, and KPC/ESBL BSI, respectively. A mean bed-day cost of 132€ was estimated.

### 3.1. Cost-Effectiveness Analysis

According to the cost-effectiveness analysis, FQP was the dominating strategy. Thus, no incremental cost-effectiveness ratios (ICERs) were calculated. The full results of the analysis are shown in Table 2.

### 3.2. Sensitivity Analysis

The results of the one-way sensitivity analyses are summarized in Table 3. The difference in costs between FQP vs. no prophylaxis ranged between 18.36 and 87.74€ saved per patient. The difference in effects between FQP vs. no prophylaxis ranged between −0.11 and 0.24 LYs gained. Considering no FQP vs. FQP, the difference in costs ranged between additional 33.61 and 80.59€ per patient, whereas the difference in effects ranged between 0.11 and 0.03 LYs lost (around 40 and 11 days), Table 3.

## 4. Discussion

This study evaluated the costs and effects associated with two alternative strategies (FQP vs. no prophylaxis) for patients with hematological malignancies receiving transplantation. According to the cost-effectiveness analysis, FQP was the dominating strategy. The difference in costs in FQP ranged between 18.36 and 87.74€ saved per patient and the difference in effects ranged between −0.11 and 0.24 LYs gained at one-way sensitivity analysis. Considering no FQP, the difference in costs ranged between additional 33.61 and 80.59€ per patient, whereas the difference in effects ranged between 0.11 and 0.03 LYs lost (around 40 and 11 days).

Although the use of FQ increases the risk of colonization with FQ resistant strains, inadequate data and confounding variables associated with local epidemiology hinder estimating the risk. Currently, Infectious Disease Society of America (IDSA) guidelines do not recommend the routine use of prophylaxis in neutropenic patients [13]. The recommendation branches from two basic concerns: the emerging drug-resistant organisms due to broad antibiotic use and the fact that prophylaxis was not associated with decreased mortality, despite sufficient evidence supporting its effectiveness in decreasing febrile episodes.

According to the national surveillance of antimicrobial resistance in Italy in 2021, the resistance of *E. coli* to fluoroquinolones decreased from 44.4% in 2015 to 32.5% in 2021. Similarly, resistance to fluoroquinolones by *Klebsiella pneumoniae* carbapenemase decreased from 61% in 2015 to 50.1% in 2021 [26,27]. In our study, we also performed a sensitivity analysis over the 95% range for resistance input parameters to account for uncertainty.

Regarding cost-utility, in-line with our results levofloxacin prophylaxis, compared to no prophylaxis, was cost-effective in decreasing bacterial sepsis in children receiving chemotherapy for acute myeloid leukemia or relapsed acute lymphocytic leukemia. The prophylaxis was also shown to be cost saving when the costs of infectious complications and further adverse events are considered [28,29].

Pagano et al. reported in the Hema e-Chart registry that FQP may reduce the necessity for empiric antibiotic therapy, and consequently exposure to broad spectrum antibiotics. However, since the majority of the patients receiving FQP still manifest fever during neutropenia [30], the benefit may be of slight clinical significance. Other approaches, such as early cessation or de-escalation of empiric therapy when appropriate, could be more effective in restricting the exposure to broad spectrum antibiotics [11].

Bucaneve et al. evaluated in a large study the efficacy of FQs prophylaxis in a population with a 20% resistance rate to FQs in gram-negatives. The study suggested that FQ prophylaxis should be considered in locations with similar or lower resistance rates. Levofloxacin was effective in preventing febrile episodes and infections in cancer patients with and without neutropenia. However, the long-term effect of this intervention on resistance was not outlined [17,19,31]. In a meta-analysis of ninety-five trials, fluoroquinolone prophylaxis was studied in neutropenic patients. It has reduced the overall mortality and infection-related mortality, fever and infections rates. However, it has also increased the risk for harboring a subsequent bacilli resistance to the drug but these results were not statistically significant [17].

On the other hand, Caldwell et al. showed that no FQP in an hemato-oncology setting resulted in significantly lower rates of resistant *E. coli* isolates in BSI, without a significant increase in mortality, ICU admissions or length of stay. However, the sample size was small and limited to a single organism, though the results support the cessation of FQP. Earlier randomized controlled trials had examined antimicrobial prophylaxis in cancer patients. The results reported reductions in fever episodes but not mortality [18,19]. Caldwell et al. suggest that discarding FQP might result in increased febrile episodes but later in decreased resistant isolates, along with no increase in mortality or ICU admission [32].

Infections caused by AMR organisms are difficult to treat, increasing costs and adverse events to common therapeutic regimens. Knowledge is still evolving to combat resistance mechanisms influenced by inappropriate prescription, misuse of antibiotics, and improper diagnosis. Current forces are being directed to prevent a “post-antibiotic era” where minor infections become a leading cause of death. Various approaches include: appropriate prophylaxis, combination therapies, synthetized antimicrobials, active surveillance and comprehension of mechanisms of resistance to prevail AMR threats. Therefore, a multi-disciplinary collaboration is crucial to fulfill effective measures to control this crisis [33].

Given the small differences in terms of costs and effects between the two strategies in our highly endemic setting [34], no prophylaxis seems an appropriate choice. However, this analysis did not consider the broader effect on hospital ecology of multiple doses of FQP, which could provide further support for the strategy of no prophylaxis. Other limitations of our study are the comparison of two different periods and strategies which include various case mix of the admitted patients over time. Moreover, costs are probably underestimated, as a result of considering costs due to hospital stay and prophylaxis, rather than treatment and laboratory costs. As any model, we were also constrained by the relatively limited data and by the validity of our assumptions.

Therefore, the decision of FQP should be in line with national stewardship programs and local epidemiological practices [35,36]. Although no robust cut off for the efficacy of FQP could be outlined yet [33], the global crisis of antimicrobial resistance calls for adjusting the established indications [37,38]. Future studies should investigate the long-term impact of prophylaxis on patterns of resistance.

## 5. Conclusions

Given the small differences in terms of costs and effects between the two strategies, no prophylaxis seems an appropriate choice. Furthermore, this analysis did not consider the broader effect on hospital ecology of multiple doses of FQP, which could provide further support for the strategy of no prophylaxis. Our results suggest that the necessity for FQP in an onco-hematologic setting should be determined based on local antibiotic resistance patterns. Resistance can be controlled by using antibiotics prudently under coordinated activities and stewardship guidelines to preserve novel drugs for future generations.

## Figures and Tables

**Table 1:** Input parameters.

Input	Frequency	Percentage	95% CI
**I. Probabilities**			
FQP			
Colonization KPC/ESBL:	7	6.8%	2.78–13.5
BSI rate in colonized:			
o No BSI	4	57.97%	20.48–93.8
o Non-AMR BSI	3	42.03%	9.9–81.4
o AMR BSI	0	0	
BSI rate in not colonized:			
o No BSI	61	63.5%	53–73
o Non-AMR BSI	27	28%	19.4–38.2
o AMR BSI	8	8.3%	3.6–15.7
No prophylaxis			
Colonization KPC/ESBL:	62	29.18%	23.33–36.03
BSI rate in colonized:			
o No BSI	25	40%	28–53.5
o Non-AMR BSI	19	30.6%	19.5–43.6
o AMR BSI	18	29%	18.2–41.9
BSI rate in not colonized:			
o No BSI	79	53%	44.6–61.2
o Non-AMR BSI	66	44%	36.17–52.6
o AMR BSI	4	2.6%	0.74–6.7
Mortality rate			
o No BSI	67	20.81%	15.02–27.63
o BSI	82	25.49%	18.8–33.16
o AMR BSI	111	34.38%	18.57–53.19
**II. Costs**			
Length of stay	Median (days)		IQR
o No BSI	32		29–37.75
o BSI	35		30–44
o AMR BSI	35.5		32–43
o Bed-day cost	Mean (132€)		IQR
o No BSI	4224		3828–4983
o BSI	4620		3960–5808
o AMR BSI	4686		4224–5676
Surgical prophylaxis	0.57€		
**III. Effects**			
3% discounting	8.42 LY gained		

AMR: antimicrobial resistance; BSI: bloodstream infections; ESBL: extended-spectrum beta lactamase; FQP: fluoroquinolone prophylaxis; KPC: *Klebsiella pneumoniae* carbapenemase.

**Table 2:** Base case scenario: costs and effects associated with both strategies.

Strategy	Cost, €	Incremental Cost, €	Effect (Life-Years Gained)	Incremental Effect (Life-Years Gained)	C/E	INCREMENTAL C/E (ICER per LY Gained)
FQP	4375.26	56	6.46		677.28	Dominated
No FQP	4431.26		6.39	−0.07	693.47	Dominant

FQP: fluoroquinolone prophylaxis.

**Table 3:** Range of results of the one-way sensitivity analysis.

Strategy	Range Cost, €	Range Incremental Cost	Range Effect (Life-Years Gained)	Range Incremental Effect
FQP	4343.52–4412.9	−87.74–−18.36	6.28–6.63	−0.11–0.24
No FQP	4408.87–4455.85	33.61–80.59	6.35–6.43	−0.11–−0.03

FQP: fluoroquinolone prophylaxis.

## Data Availability

Data is available upon request to the corresponding author.
